# Changes of biomarkers for erythropoiesis, iron metabolism, and FGF23 by supplementation with roxadustat in patients on hemodialysis

**DOI:** 10.1038/s41598-023-30331-6

**Published:** 2023-02-23

**Authors:** Shunsuke Yoshida, Tomohiro Saito, Keigo Shibagaki, Keiichi Hirao, Takatoshi Yuza, Naohisa Tomosugi, Hirokazu Honda

**Affiliations:** 1 Division of Nephrology, Department of Medicine, Showa University School of Medicine, 1-5-8 Hatanodai, Shinagawa-ku, Tokyo, 142-8666 Japan; 2 Shibagaki Clinic Jiyugaoka, Tokyo, Japan; 3 Shibagaki Clinic Togoshi, Tokyo, Japan; 4 Shibagaki Clinic Kugahara, Tokyo, Japan; 5 Division of Systems Bioscience for Drug Discovery, Medical Research Institute, Kanazawa Medical University, Kanazawa, Japan

**Keywords:** Nephrology, Medical research

## Abstract

This study aimed to confirm changes in biomarkers of erythropoiesis and iron metabolism and serum fibroblast growth factor 23 (FGF-23) during darbepoetin-α treatment and then switching to the hypoxia-inducible factor prolyl hydroxylase inhibitor roxadustat. A total of 28 patients on hemodialysis who received weekly doses of darbepoetin-α were switched to roxadustat. Biomarkers for erythropoiesis and iron metabolism and intact and C-terminal FGF-23 were measured in blood samples collected before the HD session on days − 7 (darbepoetin-α injection), − 4, and − 2, and days 0 (switch to roxadustat treatment, three times weekly), 3, 5, 7, 14, 21, and 28. Erythropoietin and erythroferrone levels were elevated on day − 4 by darbepoetin-α injection and decreased to baseline levels at day 0. Levels of erythropoietin were not significantly increased by roxadustat supplementation, but erythroferrone levels were continuously elevated, similar to darbepoetin-α treatment. Hepcidin-25 and total iron binding capacity were significantly decreased or increased in patients treated with roxadustat compared with darbepoetin-α. Changes of intact and C-terminal FGF-23 levels were parallel to changes of phosphate levels during roxadustat treatment. However, the actual and percentage changes of intact FGF-23 and C-terminal FGF-23 in patients with low ferritin levels were greater than those in patients with high ferritin levels. Roxadustat might stimulate erythropoiesis by increasing iron usage through hepcidin-25, which was suppressed by erythroferrone in the physiological erythropoietin condition. Changes of intact FGF-23 and C-terminal FGF-23 levels might be affected by roxadustat in patients on hemodialysis, especially those with a low-iron condition.

## Introduction

Hypoxia-inducible factor prolyl hydroxylase (HIF-PH) inhibitors are characterized by physiological levels of endogenous erythropoietin production and improved iron metabolism, different from the effect of erythropoiesis-stimulating agents (ESAs) on erythropoiesis. ESA treatment induces erythropoiesis by a high dose of exogenous EPO supplementation that indirectly increases iron metabolism in the course of erythropoiesis. On the other hand, HIF-PH inhibitors directly induce transporter of iron absorption (divalent metal transporter 1), iron exporter from cells (ferroportin), and carrier protein for iron delivery (transferrin), thus improving iron metabolism^1,2^. Moreover, HIF-PH inhibitors lower levels of hepcidin-25 in non-dialysis-dependent patients and hemodialysis patients more than ESA treatment^3–6^.

Furthermore, HIF-PH inhibitors influence fibroblast growth factor 23 (FGF-23) metabolism^7,8^. FGF-23 is secreted mainly by osteocytes and is a key regulator of serum phosphate levels. FGF-23 production and cleavage are influenced by not only phosphate levels, but also by iron deficiency, chronic inflammation^9,10^, and EPO^11^. Clinkenbeard et al. confirmed that recombinant human EPO (rHuEPO), a short-acting ESA, might induce the expression of bone FGF-23 production in vivo and increase serum intact FGF-23 levels in patients with anemia^11^. The effect of long-acting ESA on FGF-23 metabolism might differ from that of rHuEPO and could increase cleavage of FGF, decreasing intact FGF-23 and increasing C-terminal FGF-23 due to hepcidin-25 suppression^12^. HIF-PH inhibitors also increased FGF-23 due to EPO production, but the increased levels of FGF-23 were quite low compared with those with EPO treatment^7^. The effects of HIF-PH inhibitors on FGF-23 are similar to those of long-acting ESAs, and HIF-PH inhibitors could increase cleavage of FGF-23, decreasing intact FGF-23^8^. As mentioned above, HIF-PH inhibitors decrease hepcidin-25 more than ESAs^3–6^; thus, HIF-PH inhibitors might potentially decrease intact FGF-23 more than long-term ESA treatment in clinical settings.

However, HIF does not decrease hepcidin-25 directly^2,13^, and the behaviors of hepcidin-25 and the biomarkers that regulate hepcidin-25 metabolism with HIF-PH inhibitor treatment are unclear. Therefore, the aim of the present study was to confirm the changes in and behaviors of biomarkers of erythropoiesis and iron metabolism by the HIF-PH inhibitor, roxadustat, compared with those by a long-acting ESA, darbepoetin-α (DA), and then examine whether the effects of the HIF-PH inhibitor on serum FGF-23 values would be similar to those of ESA considering the conditions of stored iron in patients on HD.

## Results

### Baseline characteristics and changes in roxadustat doses

Table 1 shows the patients’ characteristics at baseline (day − 7). All enrolled patients were anuric. Iron therapy was given to 64% of patients, of which 6 patients (21%) received intravenous iron therapy. The doses and frequencies of intravenous and oral iron therapy were not changed during the study period. No patients were started on iron therapy in this period.

**Table 1:** Patient characteristics and laboratory data at baseline.

Number of patients	28
Age (year)	75 ± 12
Gender (% of men)	43
Body mass index (kg/m^2^)	21.2 ± 3.9
Diabetes mellitus (%)	14
Hypertension (%)	100
Primary disease (%)	
Nephrosclerosis	36
Chronic glomerulonephritis	21
Diabetic nephropathy	7
Others	7
Unknown	29
Dialysis vintage (months)	76 (37, 129)
Kt/V	1.7 ± 0.3
Darbepoetin-α dose (μg)	19 ± 11
Iron therapy (%)	64
Intravenous iron (N, %)	6, 21
Oral iron (N, %)	43
Ferric citrate hydrate (N, mean doses (mg/day))	8, 1000 ± 194
Sodium ferrous citrate (N, mean doses (mg/day))	3, 66.7 ± 28.9
Red blood cell count (/μL)	374 ± 40
Hemoglobin (g/dL)	11.5 ± 0.7
Mean corpuscular hemoglobin (pg)	30.4 ± 2.5
Mean corpuscular volume (fL)	97.9 ± 6.9
Reticulocyte count (10^4^/μL)	5.0 ± 1.5
Red blood cell distribution width-CV (%)	14.3 ± 1.1
Platelet (× 10^4^/μL)	20.3 ± 6.8
Albumin (g/dL)	3.7 ± 0.3
Phosphate (mg/dL)	5.0 ± 1.1
Albumin adjusted calcium (mg/dL)	9.0 ± 0.6
High sensitive C-reactive protein (mg/dL)	0.06 (0.05, 0.22)
Iron (μg/dL)	69.6 ± 23.4
Total iron binding capacity (μg/dL)	244 ± 30.4
Transferrin saturation (%)	28.8 ± 10.2
Ferritin (ng/mL)	130.6 (65.2, 154.2)
Hepcidin-25 (ng/mL)	46.0 ± 26.8
Erythropoietin (mIU/mL)	9.5 (8.0, 14.5)
Erythroferrone (ng/mL)	0.26 (0.13, 0.74)
Growth differentiation factor 15 (pg/mL)	5282 (4444, 6787)
Intact parathyroid hormone (pg/mL)	183 (145, 276)
Intact fibroblast growth factor 23 (pg/mL)	1228 ± 852
C-terminal fibroblast growth factor 23 (RU/mL)	711 ± 511

*CV* coefficient of variation.

The mean dose of DA on day − 7 was 19 ± 11 μg, and 64% of patients received a phosphate binder containing iron or received intravenous iron therapy. Mean doses of roxadustat (per day) were 70 mg for the first two weeks, 68.6 ± 5.2 mg in the 3rd week, and 63.2 ± 12.2 mg in the 4th week.

### Changes in parameters of erythropoiesis and iron metabolism with DA treatment and after switching to roxadustat supplementation

**Figure 1:**
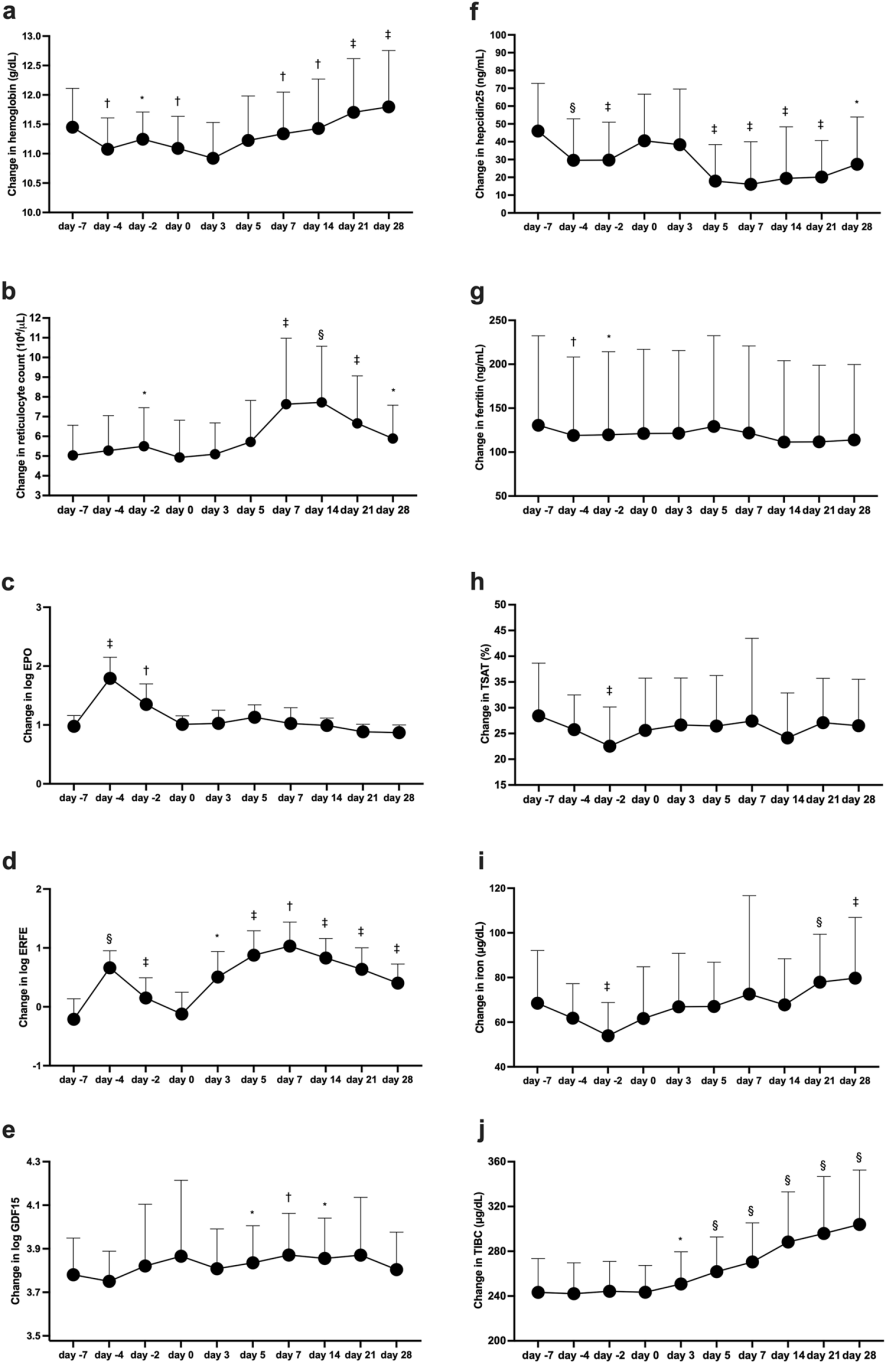
Changes in parameters of erythropoiesis and iron metabolism by darbepoetin-α treatment and roxadustat treatment. Changes in levels of hemoglobin (**a**), reticulocyte counts (**b**), erythropoietin (EPO) (**c**), erythroferrone (ERFE) (**d**), growth differentiation factor 15 (GDF-15) (**e**), hepcidin-25 (**f**), ferritin (**g**), transferrin saturation (TSAT) (**h**), iron (**i**), and total iron binding capacity (TIBC) (**j**). Darbepoetin-α (DA) is injected at day − 7, and roxadustat supplementation is started from day 0. Data during DA treatment are compared between the value at day − 7 and the target value, and data during roxadustat treatment are compared between the value at day 0 and the target value. Data are shown as means ± standard deviation. *p < 0.05, ^†^p < 0.01, ^‡^p < 0.001, ^§^p < 0.0001.

Hemoglobin levels were changed to between 11.0 and 11.5 g/dl during DA treatment (Fig. 1a), and changes in reticulocyte counts were similar to those in hemoglobin (Fig. 1b). Levels of EPO and erythroferrone were significantly elevated at day − 4 and day − 2 by DA treatment (Fig. 1c,d and Supplementary Fig. S1a–d), whereas GDF-15 levels at day − 4 did not change from baseline (Fig. 1e). Hepcidin-25 levels were significantly decreased from approximately 45–25 ng/mL by DA treatment (Fig. 1f), and changes in ferritin were similar to those in hepcidin-25 (Fig. 1g). Changes in transferrin saturation were similar to those in iron (Fig. 1h,i).

**Figure 2:**
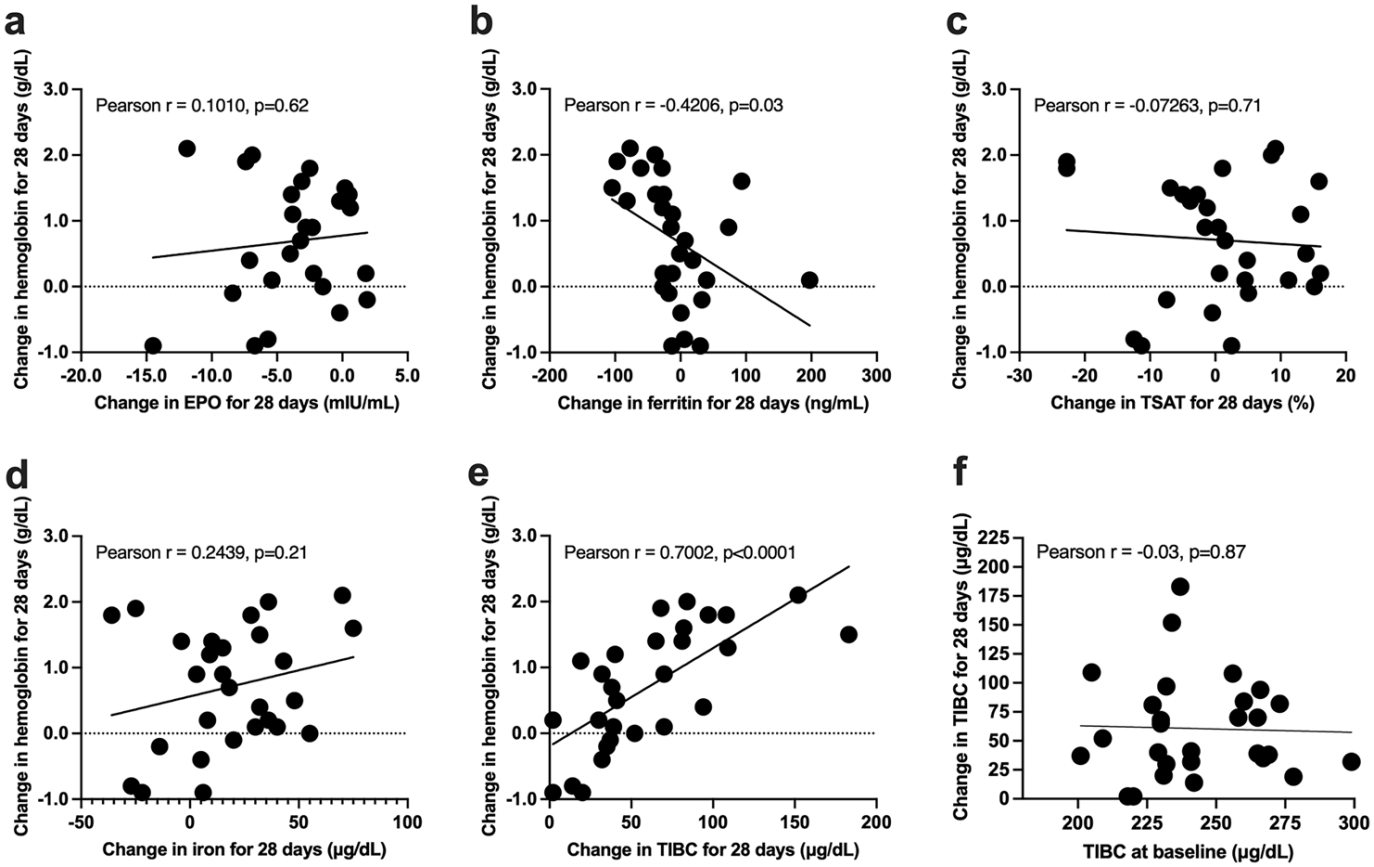
Associations among changes in hemoglobin and erythropoietin or parameters of iron metabolism, and between changes in total iron binding capacity and total iron binding capacity at day 0 during roxadustat treatment. Associations of changes in hemoglobin for 28 days with changes in erythropoietin (**a**), ferritin (**b**), transferrin saturation (TSAT) (**c**), iron (**d**), total iron binding capacity (TIBC) (**e**) for 28 days, and changes in TIBC for 28 days and TIBC at day 0 (**f**).

At day 0, DA treatment was switched to roxadustat supplementation. Levels of hemoglobin and reticulocyte counts were increased by roxadustat supplementation (Fig. 1a,b). EPO levels did not increase from day 0 to other days from day 3 to day 28 (Fig. 1c and Supplementary Fig. S1d), but erythroferrone levels were significantly elevated from day 3 to day 28 compared to those at day 0 (Fig. 1d). Changes in GDF-15 levels fluctuated, and the levels on day 5 or days 7 and 14 were decreased or increased by roxadustat treatment, respectively, compared with the levels on day 0 (Fig. 1e). Hepcidin-25 levels were significantly decreased from day 5 to day 28; they were decreased and suppressed more than by DA (Fig. 1f). Changes in ferritin tended to be decreased from day 7 to day 28 (Fig. 1g). Increases in hemoglobin by roxadustat supplementation were significantly associated with changes in ferritin and TIBC (Figs. 1j, 2).

Levels of hepcidin-25 and ferritin in patients given intravenous and oral iron were not significantly different from those in patients who were not given iron on day 0, day 7, day 14, and day 21 (Supplementary Table S1). Doses of ferric citrate hydrate were positively associated with levels of hepcidin-25 on day 7, day 14, day 21 and day 28 (Supplementary Table S1).

### Changes in intact and C-terminal FGF23 levels by DA treatment and after switching to roxadustat supplementation

Levels of intact and C-terminal FGF23 in patients given intravenous and oral iron were not significantly different from those who were not given iron on day 0, day 7, day 14, day 21 and day 28 (Supplementary Table S1). Doses of ferric citrate hydrate were not associated with levels of intact and C-terminal FGF23 on day 0, day 7, day 14, day 21 and day 28 (Supplementary Table S1).

**Figure 3:**
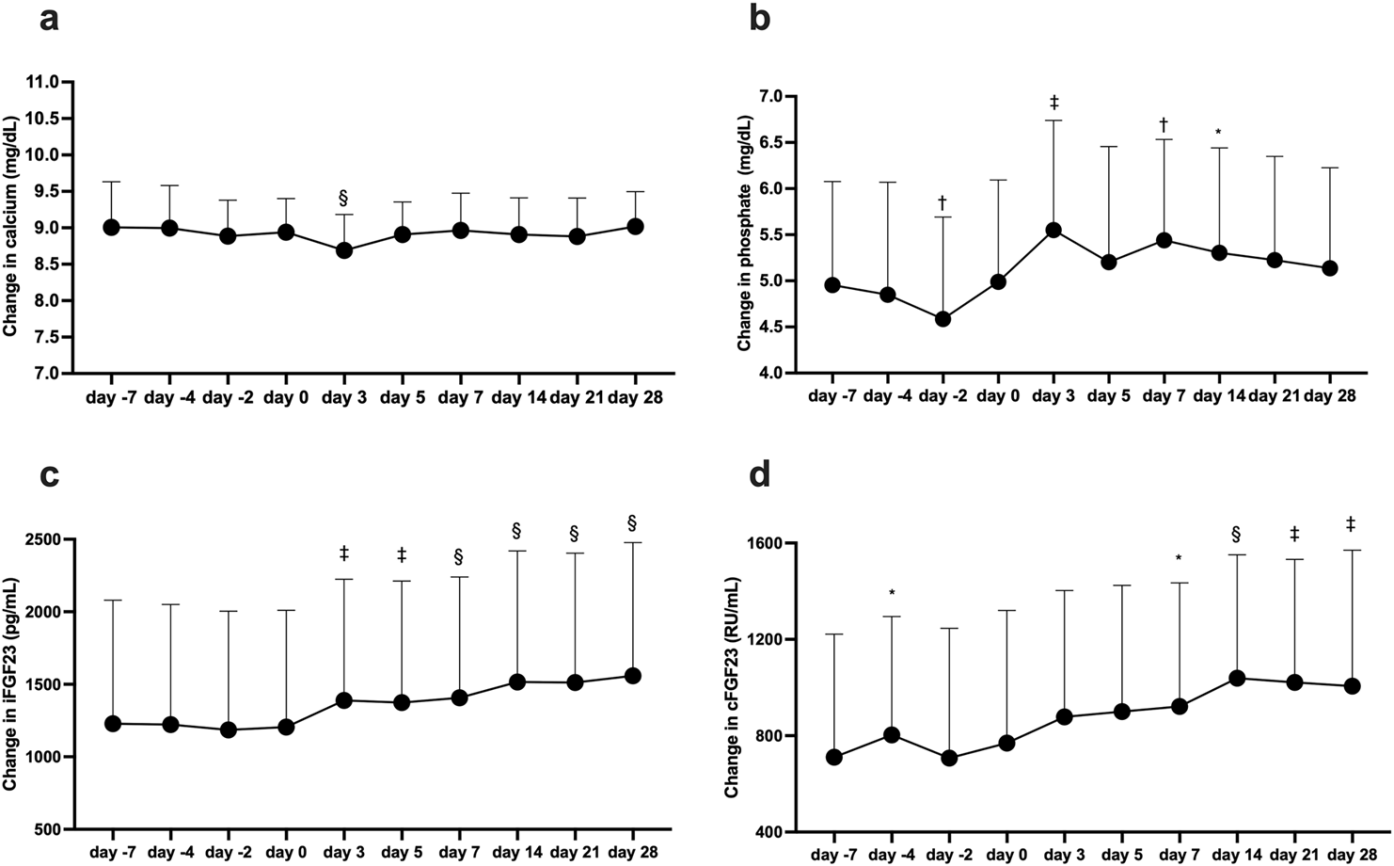
Changes in calcium, phosphate, and intact and C-terminal fibroblast growth factor 23 (FGF-23) levels. Changes in albumin-adjusted calcium (**a**), phosphate (**b**), and intact FGF23 (iFGF23) (**c**) and C-terminal FGF23 (cFGF-23) (**d**) levels after darbepoetin-α injection (at day − 7), and roxadustat supplementation (started from day 0). Data are shown as means ± standard deviation. Data during DA treatment are compared between the value at day − 7 and the target value, and data during roxadustat treatment are compared between the value at day 0 and the target value. *p < 0.05, ^†^p < 0.01, ^‡^p < 0.001, ^§^p < 0.0001.

Calcium levels were transiently lower on day 3 than on day 0 (Fig. 3a). Levels of phosphate were increased from day 3 during the study period (Fig. 3b), and the changes were correlated with changes in intact and C-terminal FGF-23, which were elevated from day 3 (Fig. 3c,d).

**Figure 4:**
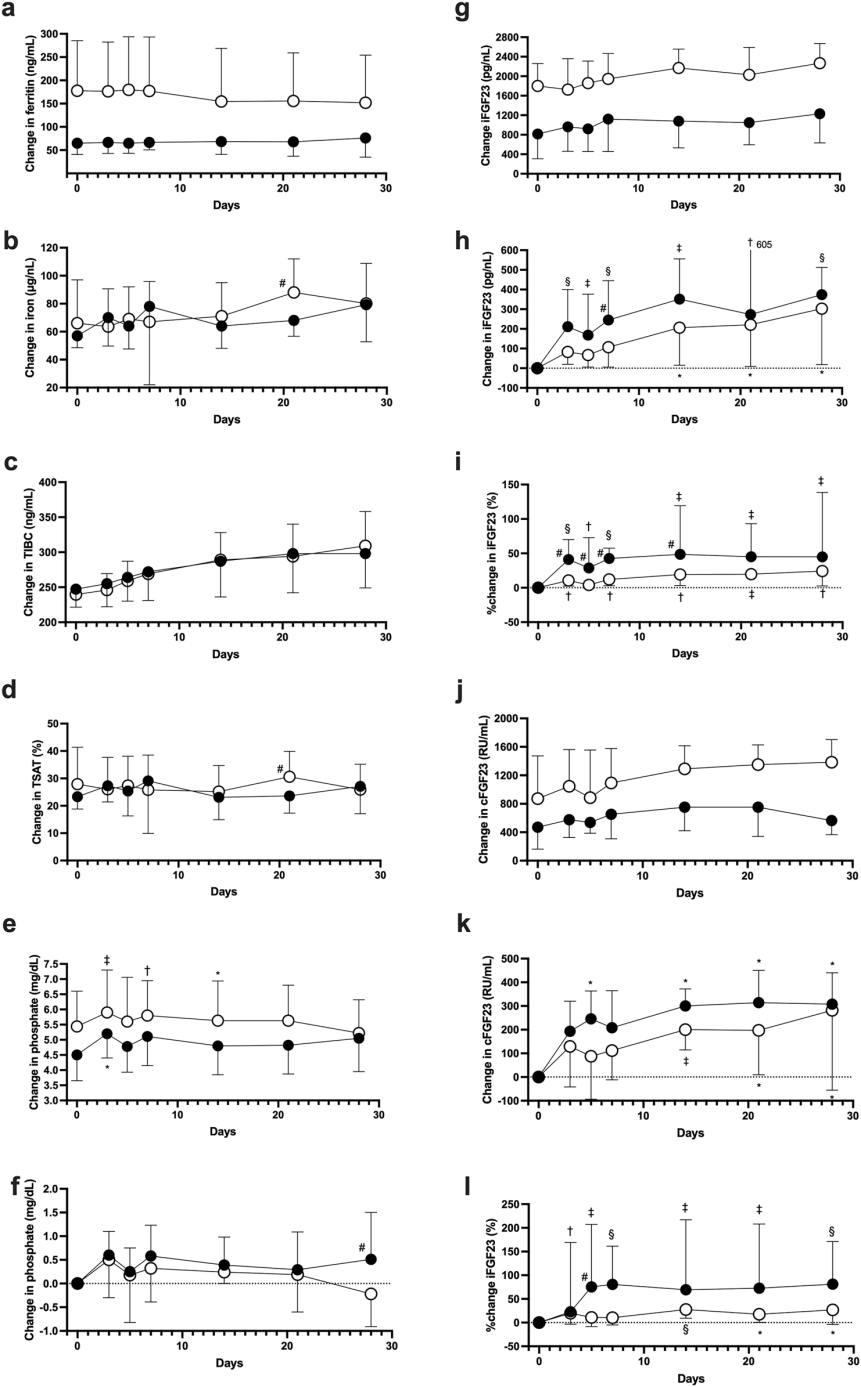
Changes in ferritin, iron, total iron binding capacity (TIBC), transferrin saturation (TSAT), phosphate, and intact and C-terminal fibroblast growth factor 23 (FGF23) levels grouped by the ferritin level with roxadustat treatment. Changes in ferritin (**a**), iron (**b**), TIBC (**c**), TSAT (**d**), phosphate (**e**), delta phosphate (**f**), intact FGF23 (iFGF23) (**g**), delta FGF23 (**h**), C-terminal FGF23 (cFGF23) (**j**), delta cFGF23, and percentage changes in iFGF23 (**i**) and cFGF-23 (**l**) grouped by ferritin < 100 (closed circle) or ferritin ≥ 100 ng/mL (open circle) during roxadustat treatment. Data are shown as means ± standard deviation (**a**–**f**) or medians (interquartile range) (**g**–**l**) and compared between the value at day 0 and the target value. *p < 0.05, ^†^p < 0.01, ^‡^p < 0.001, ^§^p < 0.0001. ^#^Means significant differences between the groups.

To confirm whether the effect of phosphate on FGF-23 metabolism differed with the HIF-PH inhibitor in the condition between low and adequate iron stores, changes in FGF-23 were assessed according to low (ferritin < 100 ng/mL; n = 14) or adequate levels of ferritin (ferritin ≥ 100 ng/mL; n = 14) (Supplementary Table S2, Fig. 4). Levels of iron, TIBC, TSAT, and EPO did not differ between the adequate and low-ferritin groups (Supplementary Table S2, Fig. 4b–d, Supplementary Fig. S4a). Levels of erythroferrone were more elevated in the adequate ferritin group than in the low-ferritin group, and levels of GDF15 in the adequate ferritin group were transiently elevated from day 5 to day 7 (Supplementary Fig. S4b,c). Changes in hepcidin-25 levels in the low-ferritin group were similar to those in the adequate ferritin group (Supplementary Fig. S4d). Phosphate levels were higher in the adequate ferritin group than in the low-ferritin group (Supplementary Table S2, Fig. 4e), but changes in phosphate from day 0 to day 21 were of a similar degree in the two groups (Fig. 4f, Supplementary Fig. S4e). Levels of intact FGF-23 were significantly higher in the adequate ferritin group than in the low-ferritin group (Supplementary Table S2, Fig. 4g). Values of intact FGF-23 in both groups were elevated along with changes in phosphate (Fig. 4e,g). However, the changes in intact FGF-23 in the adequate ferritin group were mild compared with those in the low-ferritin group (Fig. 4h,i); nevertheless, phosphate levels were higher in the adequate ferritin group than in the low-ferritin group (Fig. 4e). Changes in C-terminal FGF-23 levels in both groups were similar to the changes in intact FGF-23 (Fig. 4j,k), whereas the percentage change of C-terminal FGF-23 was greater in the low-ferritin group than in the adequate ferritin group (Fig. 4l).

## Discussion

This clinical study investigated changes in biomarkers of erythropoiesis and iron metabolism, as well as changes in FGF-23, associated with DA and roxadustat treatment. Erythropoiesis due to roxadustat seems to be associated with increased changes in TIBC and increased use of iron due to decreased hepcidin-25 levels. Suppression of hepcidin-25 levels by roxadustat treatment may be associated with increased erythroferrone. Changes in FGF23 are dependent on phosphate levels, but increases in FGF-23 may be prone to develop in patients on roxadustat treatment with a low-iron condition compared with those in the adequate iron condition.

Previous studies demonstrated that ESA lowered hepcidin-25 levels transiently according to the half-life of ESA^12,14,15^. In the present study, hepcidin-25 levels were more suppressed by roxadustat than by DA treatment. One reason for the significant suppression of hepcidin-25 by the HIF-PH inhibitor may be related to the continuous elevation of erythroferrone, a suppressor of hepcidin-25. Erythroferrone is produced and secreted by erythroblasts in bone marrow during erythropoiesis. In fact, roxadustat treatment enhanced erythropoiesis; reticulocyte counts and hemoglobin levels were significantly increased by roxadustat treatment compared with DA treatment. After DA administration, the levels of erythroferrone were transiently but significantly increased, and the changes in erythroferrone were similar to those in EPO. It has been reported that ESA doses were correlated with the EPO concentration, and the ESA dose and EPO concentration were associated with serum erythroferrone levels in healthy volunteers^16^. In the present study, DA doses were significantly associated with serum erythroferrone levels, but EPO concentrations after DA administration were not correlated with erythroferrone levels. Though EPO concentrations with roxadustat treatment were lower than those with DA administration, erythroferrone levels were significantly and continuously elevated up to the same levels by DA treatment. EPO is a key factor for erythroferrone production in the process of erythropoiesis, even during treatment with an HIF-PH inhibitor^17^. The reason why the association of EPO with erythroferrone by DA injection was not significant is thought to be due to the fact that concentrations of exogenous EPO supplied by DA might be much higher than those required for erythroferrone production, and erythroferrone would be produced by a physiological EPO concentration^16^.

Hanudel et al. reported that erythropoiesis could occur with an HIF-PH inhibitor in an erythroferrone-knockout model, suggesting that suppression of hepcidin-25 might be induced by factors other than erythroferrone^18^. GDF-15 is thought to be a candidate that suppresses hepcidin-25 production during increased erythropoiesis and iron deficiency^19,20^, whereas elevation of GDF-15 in subjects with iron deficiency is controversial in clinical studies^21,22^, and the clinical study with subjects at altitude failed to demonstrate significant associations among GDF-15, hepcidin-25, and the endogenous EPO concentration^23^. Ashby et al. reported that GDF-15 levels were decreased after hepcidin suppression by rHuEPO injection in healthy volunteers^24^. In the present study, continuous elevation of GDF-15 levels was not observed by DA treatment and roxadustat supplementation. GDF-15 during roxadustat treatment might not have affected the changes in hepcidin 25, and GDF-15 did not correspond to the suppression of hepcidin-25.

A previous study reported that a short-acting ESA increases levels of both intact and C-terminal FGF-23 in patients with anemia^11^, but continuous EPO stimulation by long-acting ESA might increase FGF-23 degradation and increase C-terminal FGF-23, and a greater decrease in hepcidin might be associated with increased FGF-23 cleavage and subsequently increased C-terminal FGF-23 in patients on HD^12^. The HIF-PH inhibitor could increase FGF-23 degradation^7^ and suppress intact FGF-23 in animal models^7,8,19^. In the present study, DA administration increased C-terminal FGF-23, and these findings were similar to those of the previous study^12^. Levels of intact FGF-23 and C-terminal FGF-23 with roxadustat treatment were increased from day 3 because of the increased phosphate levels in the same time period. However, the actual and percentage changes of intact FGF-23 in the group with satisfactory ferritin levels were small compared with those in the low-ferritin group, though phosphate levels in the satisfactory ferritin group were high compared with those in the low-ferritin group. The changes in delta phosphate at each point were similar between the groups; thus, the impact of phosphate on changes in FGF23 may be similar between the groups. Whereas levels of hepcidin-25 before roxadustat treatment in the adequate ferritin level group were greater than those in the low-ferritin group, the decreased levels of hepcidin-25 were similar in both groups after roxadustat treatment. Thus, levels of FGF-23 with HIF-PH inhibitor treatment would be thought to improve with the stored iron utilization and iron absorption. However, the present study could not confirm changes of serum iron status, whereas TIBC levels were increased similarly in both groups. In an in vitro study, FGF23 mRNA expression in osteocyte-like cells was increased in iron deficiency conditions, and improvement of tissue iron utilization can reduce transcription and production levels of FGF23^8^. Thus, FGF-23 mRNA expression in osteocytes in the tissue low-iron condition might be more increased by a low supply of iron than those under the tissue adequate iron condition. In a study using a CKD model, HIF-PH inhibitor treatment lowered plasma intact-FGF23 levels, whereas plasma iron levels were not changed by HIF-PH inhibitor treatment^8^. Altogether, blood iron levels might not always reflect the tissue iron status in the clinical setting. Further study with well-controlled phosphate and iron levels in the clinical setting is needed to confirm associations between FGF23 metabolism and HIF-PH inhibitor treatment.

The present findings should be interpreted with the following caveats. The number of patients was relatively small, and the observational period was short. Finally, this clinical investigation could not explain causal associations among erythroferrone, GDF-15, and hepcidin-25 and determine whether the HIF-PH inhibitor caused changes in FGF-23 metabolism.

In conclusion, HIF-PH inhibitor treatment might lead to erythropoiesis by increasing iron usage through hepcidin-25, which was suppressed by erythroferrone in the physiological EPO condition, and increased levels of TIBC, which might be associated with increased hemoglobin. HIF-PH inhibitor treatment might change levels of intact FGF-23 and C-terminal FGF-23. The elevation of FGF-23 by HIF-PH inhibitors could develop in the low-iron condition compared with in the adequate iron condition.

## Methods

### Patients

The Showa University Institutional Committee on Human Research approved the protocol of the study (approval number: 3496), which proceeded according to the Declaration of Helsinki (2017 revision). All patients provided written, informed consent to participate in this study. This study included 28 patients on maintenance HD at three outpatient dialysis clinics (Shibagaki Clinic Jiyugaoka, Shibagaki Clinic Togoshi and Shibagaki Clinic Kugahara) in Japan (UMIN000045857, 26/10/2021). Recruited patients were screened according to inclusion and exclusion criteria (Supplementary Fig. S5), and the screening period was the 4 weeks following the date that consent was obtained. Patients aged > 20 years, who were treated with weekly DA for more than 3 months, had a hemoglobin level > 9.0 g/dL, ferritin level > 100 ng/mL, and/or TSAT > 20% were included in this trial. The exclusion criteria included malignant, chronic inflammatory, or severe liver or lung diseases and on anti-inflammatory or immunosuppressive agents. Patients who showed definite iron deficiency (ferritin level < 100 ng/mL and TSAT < 20%) in the screening period were excluded.

### Administration of ESA and roxadustat

The included patients were treated with intravenous DA (Nesp; Kyowa Hakko Kirin Co. Ltd., Tokyo, Japan) once weekly. The ESA dose was administered according to the Guidelines for Renal Anemia published by the Japanese Society for Dialysis Therapy 2015^25^. DA was injected at day − 7, and then roxadustat supplementation (70 mg three times weekly) was started at baseline (day 0) instead of DA in all patients.

### Iron supplementation

In patients with iron deficiency after the study started, intravenous or oral iron treatment was started if hemoglobin levels decreased to < 10 g/dL, and serum TSAT and ferritin values reached < 20% and < 100 ng/mL, respectively^25^. Intravenous iron in the form of 40-mg doses of saccharated ferric oxide (Fesin; Nichiiko Pharmaceutical, Toyoma City, Japan) was administered once weekly at baseline (day − 7) and on days 0, 7, 14, 21, and 28. Oral iron was administered daily for the study period.

Phosphate binders of ferric citrate hydrate containing iron (Riona; Torii Pharmaceutical Co. Ltd., Tokyo, Japan) or sucroferric oxyhydroxide (Petol; Kissei Pharmaceutical Co. Ltd., Matsumoto, Japan) were administered daily for the study period, and their doses were not changed during the study period.

### Blood sampling

Venous blood was sampled before the HD session at the end of the dialysis week (day − 7) and on days − 4, − 2 (DA treatment period), and baseline (day 0), day 3, day 5, day 7, day 14, day 21, and day 28 for the roxadustat treatment period.

### Measured parameters

Routine biochemical parameters and levels of albumin, calcium, phosphate, intact parathyroid hormone, high-sensitivity C-reactive protein, biomarkers of iron metabolism (serum iron, total iron-binding capacity [TIBC], ferritin, hepcidin-25, GDF-15, and erythroferrone), and intact and C-terminal FGF-23 were measured in venous blood samples obtained at baseline. Levels of phosphate, biomarkers of iron metabolism, intact FGF-23, and C-terminal FGF-23 were measured in venous blood samples at various time points thereafter. Serum samples were immediately frozen and stored at − 80 °C. Hepcidin-25 was measured using liquid chromatography-tandem mass spectrometry^26^, and GDF-15 (Quantikine ELISA Human GDF-15 Immunoassay, R&D Systems, Minneapolis, MN, USA), erythroferrone (Intrinsic Erythroferrone ELISA Kit, Intrinsic Life Sciences, La Jolla, CA, USA), intact FGF-23 (Kainos Laboratories Inc., Tokyo, Japan), and C-terminal FGF-23 (Immutopics Inc., San Clemente, CA, USA) were assayed by ELISA kits.

### Statistical analysis

Data are presented as means ± standard deviation or as medians (interquartile range) unless otherwise stated, with significance set at* P* < 0.05. Changes in parameters between days − 7 and − 4, − 2 or days 0 and 3, 5, 7, 14, 21, and 28 were compared using the paired *t-*test or the Wilcoxon matched-pairs signed-rank test. Differences in parameters between the low and adequate ferritin groups were compared using Student’s *t-*test or the Mann–Whitney U test. Differences in parameters among patients who were administrated intravenous and oral iron and those who were not administrated were compared using the Kruskal–Wallis test. Independent associations between one continuous dependent variable and independent variables were assessed by linear regression analysis. Data were statistically analyzed using JMP Pro 16.0 (SAS Institute, Cary, NC, USA) and Prism 9.0 (GraphPad Software Inc., La Jolla, CA, USA).

## Supplementary Information

Supplementary Information 1.

Supplementary Information 2.

Supplementary Information 3.

Supplementary Information 4.

Supplementary Information 5.

Supplementary Information 6.

Supplementary Information 7.

Supplementary Information 8.

## Data Availability

The datasets generated during and/or analyzed during the current study are not publicly available due to the privacy policy concerning private information of patients, but they are available from the corresponding author on reasonable request.
