# Supplementary figures and images for: Changes of biomarkers for erythropoiesis, iron metabolism, and FGF23 by supplementation with roxadustat in patients on hemodialysis
Source: Sci Rep. 2023 Feb 23;13:3181. doi: 10.1038/s41598-023-30331-6 (PMC9950357; doi:10.1038/s41598-023-30331-6)

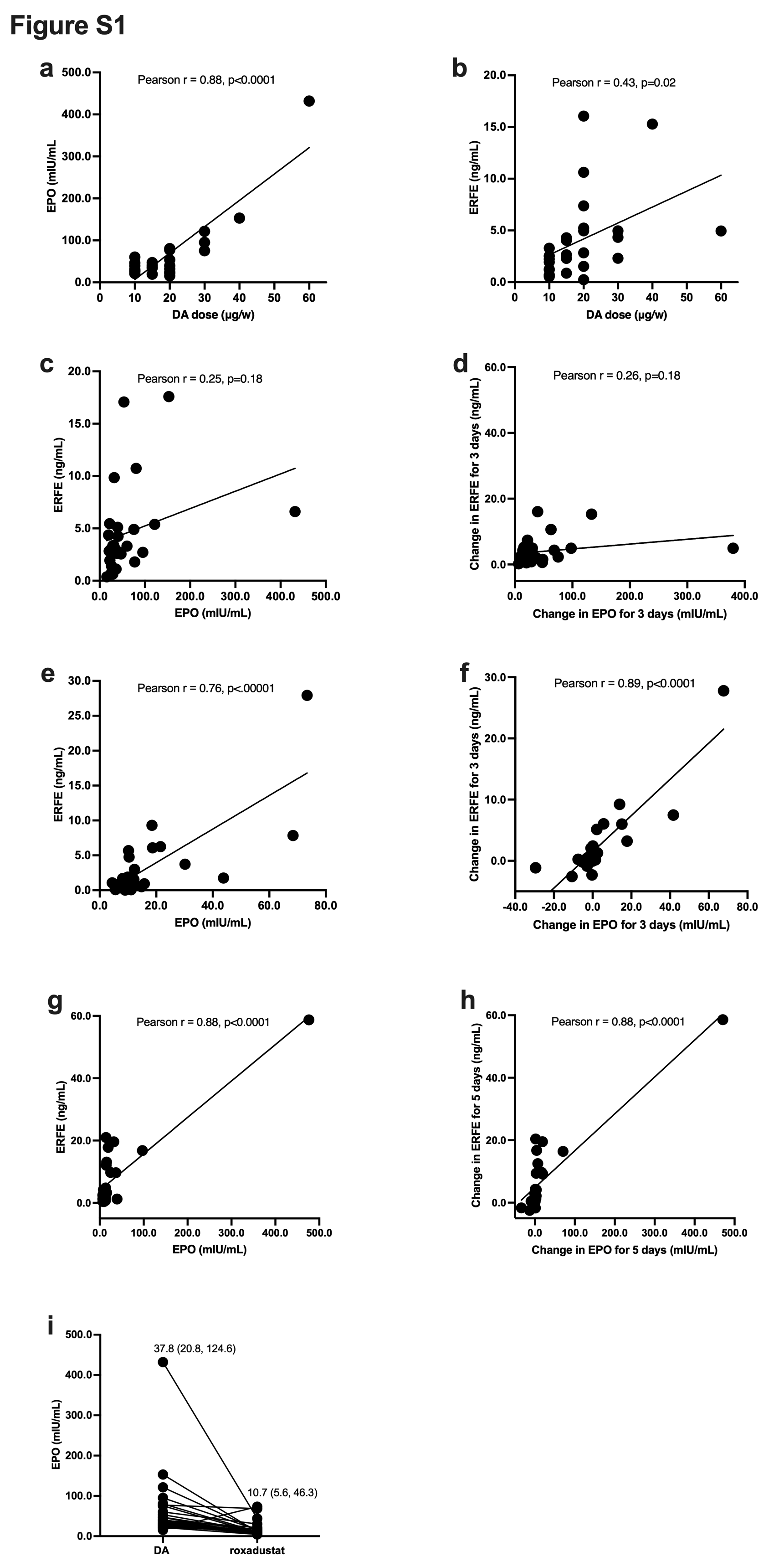

Supplement: Supplementary file 1 — Supplementary Information 1. [file 41598_2023_30331_MOESM1_ESM.tiff]

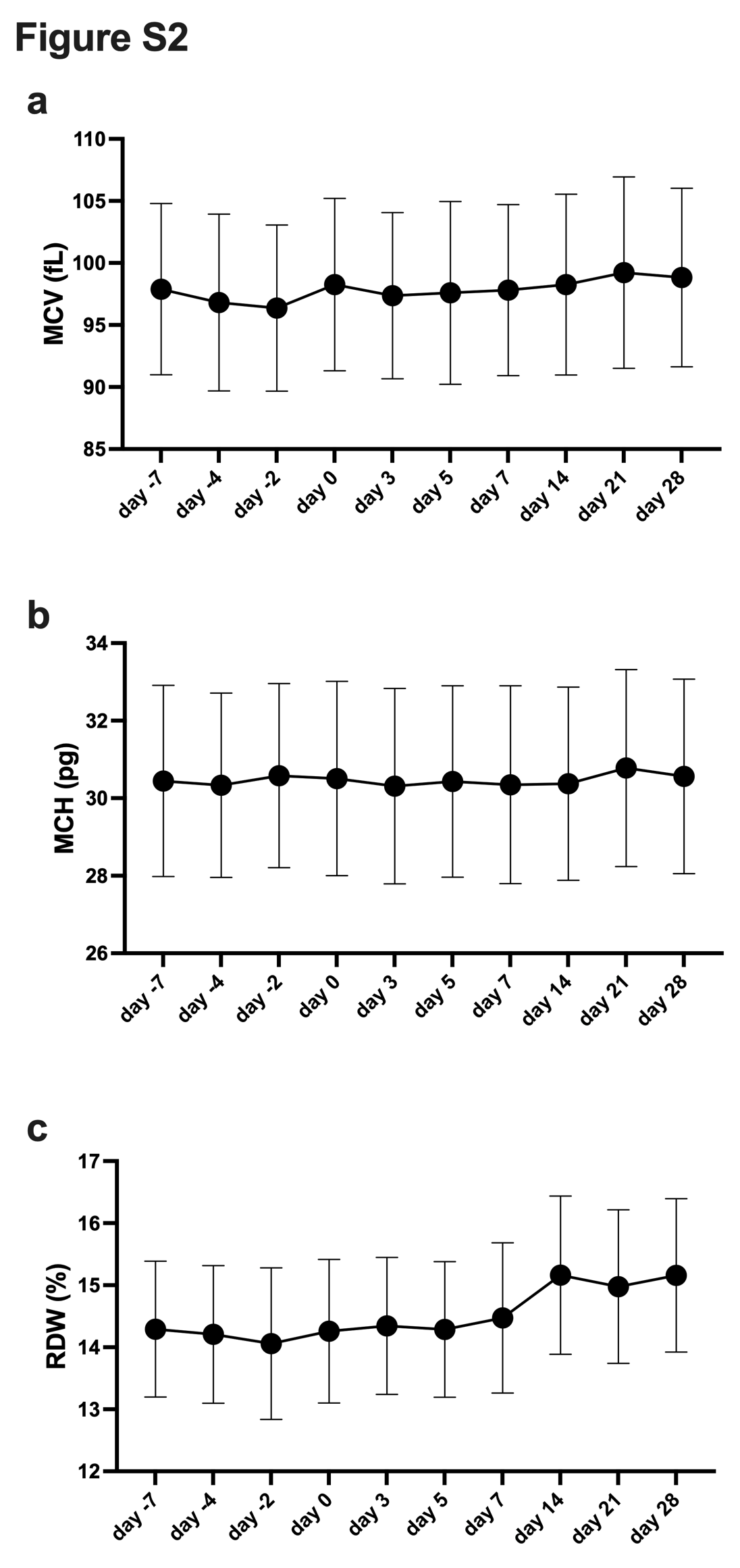

Supplement: Supplementary file 2 — Supplementary Information 2. [file 41598_2023_30331_MOESM2_ESM.tiff]

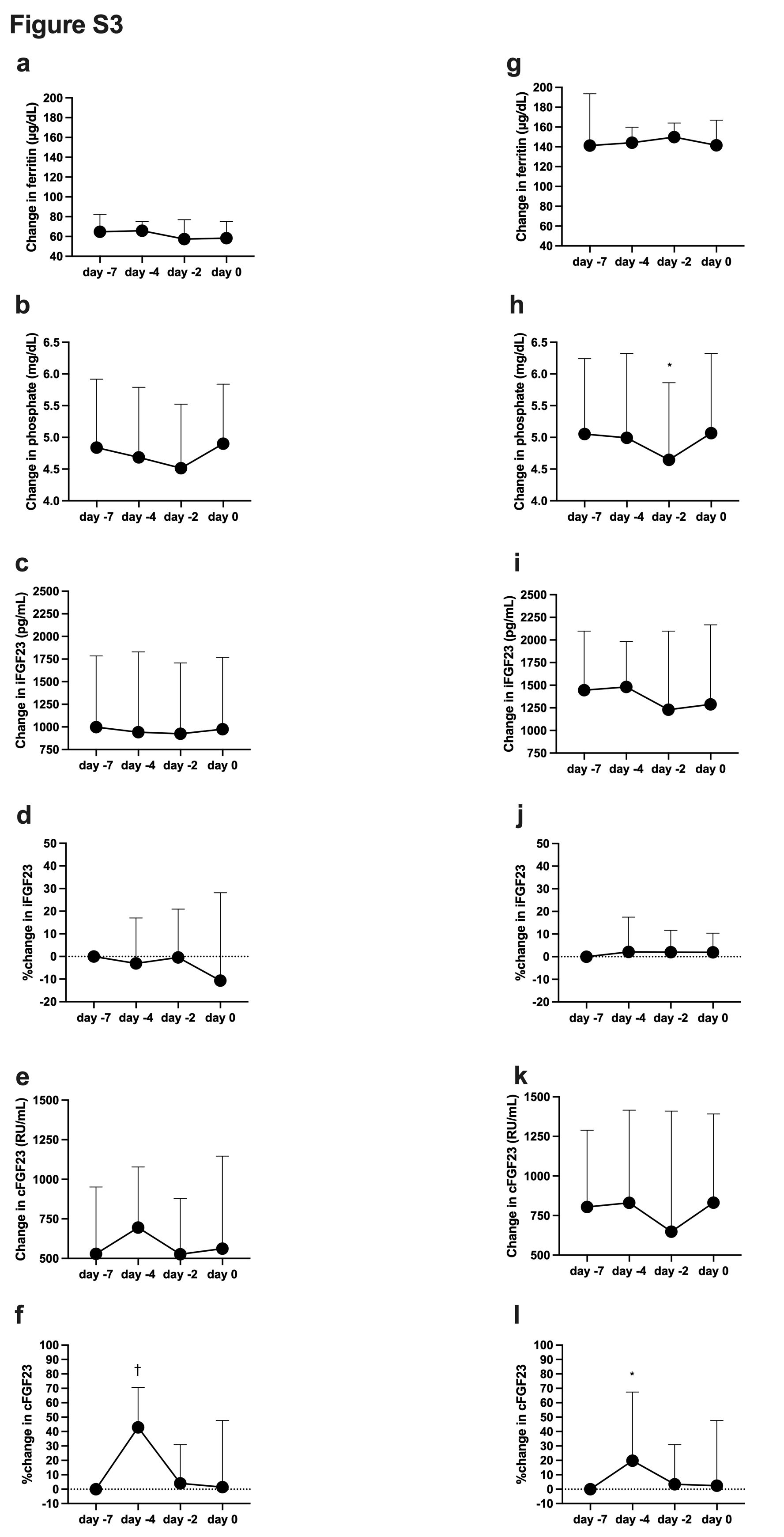

Supplement: Supplementary file 3 — Supplementary Information 3. [file 41598_2023_30331_MOESM3_ESM.tiff]

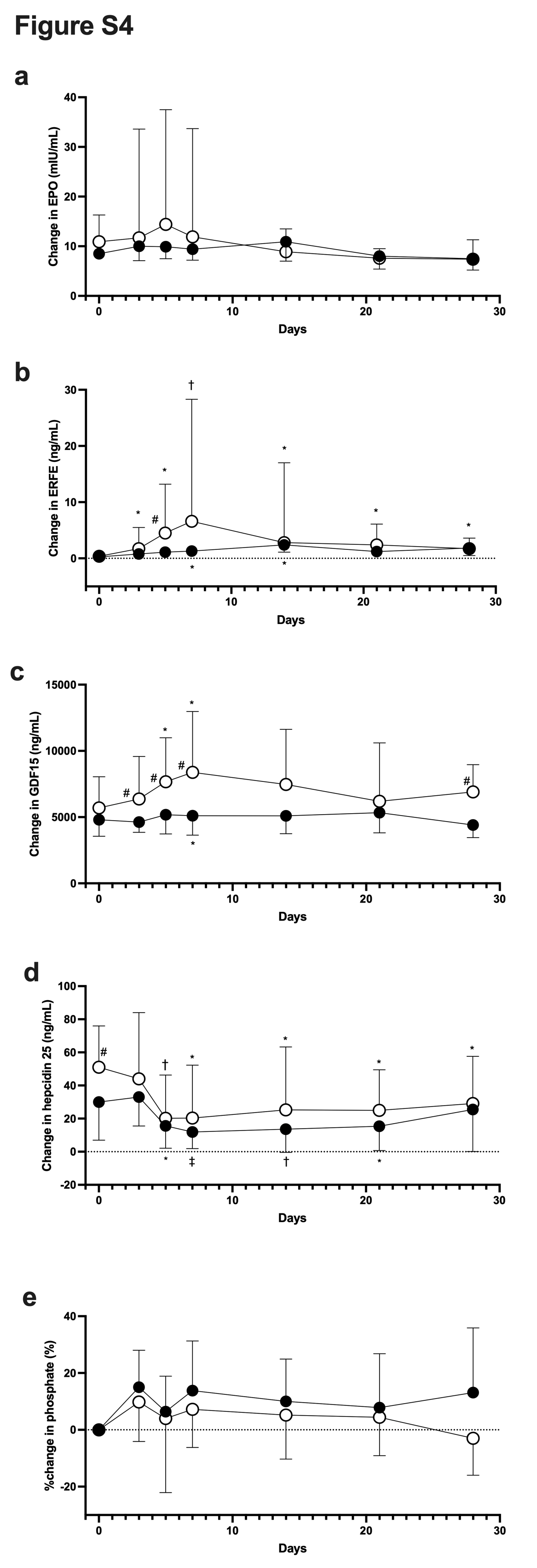

Supplement: Supplementary file 4 — Supplementary Information 4. [file 41598_2023_30331_MOESM4_ESM.tiff]

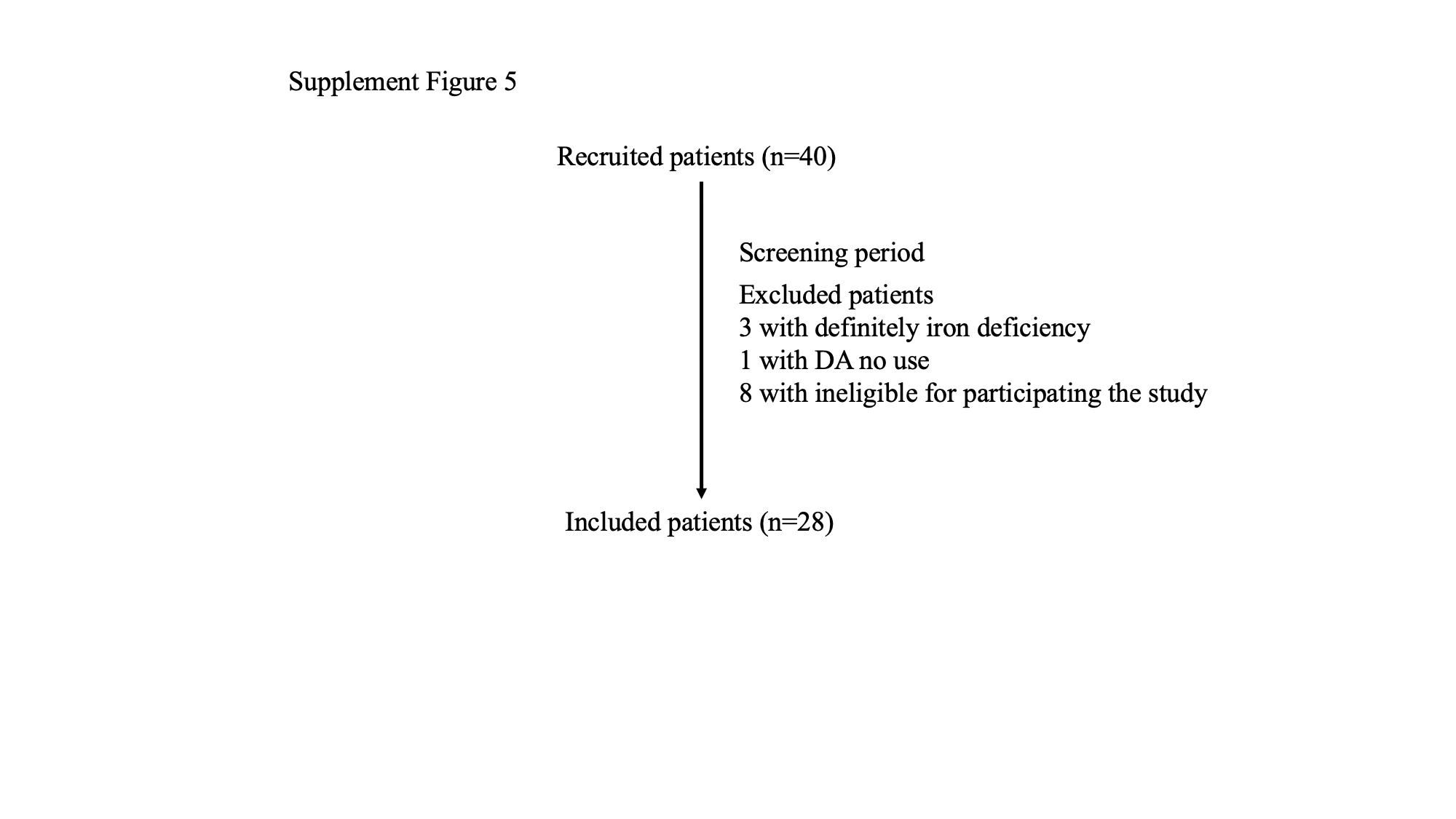

Supplement: Supplementary file 5 — Supplementary Information 5. [file 41598_2023_30331_MOESM5_ESM.tiff]
