# Supplementary material for: Changes of biomarkers for erythropoiesis, iron metabolism, and FGF23 by supplementation with roxadustat in patients on hemodialysis
Source: Sci Rep. 2023 Feb 23;13:3181. doi: 10.1038/s41598-023-30331-6 (PMC9950357; doi:10.1038/s41598-023-30331-6)
Supplement: Supplementary file 6 — Supplementary Information 6. [file 41598_2023_30331_MOESM6_ESM.docx]

Supplementary table 1. Associations of iron administration with ferritin, hepcidin-25 and FGF23.

|  | Iron therapy (intravenous, n=6; oral, n=12; none, n=10) | | | | |
| --- | --- | --- | --- | --- | --- |
|  | Ferritin (ng/mL) | | p value | Hepcidin-25 (ng/mL) | p value |
| Day 0 | 136 ± 41, 125 ± 140, 108 ± 45 | | 0.20 | 35 ± 29, 38 ± 24, 47 ± 28 | 0.76 |
| Day 7 | 129 ± 44, 130 ± 145, 107 ± 45 | | 0.18 | 5 ± 6, 24 ± 23, 12 ± 11 | 0.11 |
| Day 14 | 121 ± 65, 120 ± 130, 95 ± 46 | | 0.46 | 7 ± 7, 30 ± 39, 14 ± 16 | 0.14 |
| Day 21 | 134 ± 80, 114 ± 119, 95 ± 37 | | 0.39 | 13 ± 11, 28 ± 26, 14 ± 15 | 0.40 |
| Day 28 | 137 ± 81, 117 ± 115, 96 ± 37 | | 0.56 | 13 ± 10, 43 ± 37, 16 ± 12 | 0.006 |
|  | Iron therapy (intravenous, n=6; oral, n=12; none, n=10) | | | | |
|  | Intact FGF23 (pg/mL) | | p value | C-terminal FGF23 (RU/mL) | p value |
| Day 0 | 1580 ± 1089, 1171 ± 796, 1020 ± 612 | | 0.50 | 920 ± 672, 658 ± 531, 811 ± 529 | 0.63 |
| Day 7 | 1739 ± 1174, 1265 ± 805, 1256 ± 649 | | 0.54 | 1330 ± 426, 793 ± 513, 830 ± 472 | 0.09 |
| Day 14 | 1938 ± 1199, 1520 ± 830, 1258 ± 781 | | 0.27 | 1308 ± 480, 912 ± 522, 1028 ± 505 | 0.23 |
| Day 21 | 1962 ± 1177, 1486 ± 823, 1270 ± 765 | | 0.27 | 1381 ± 516, 984 ± 488, 848 ± 472 | 0.11 |
| Day 28 | 1953 ± 1194, 1518 ± 876, 1370 ± 806 | | 0.38 | 1229 ± 599, 904 ± 577, 993 ± 547 | 0.49 |
|  | Doses of ferric citrate hydrate (n=8) | | | | |
|  | Ferritin (ng/mL) | | | Hepcidin-25 (ng/mL) | |
|  | Pearson r | p value | | Pearson r | p value |
| Day 0 | 0.42 | 0.02 | | 0.18 | 0.35 |
| Day 7 | 0.49 | 0.008 | | 0.64 | 0.002 |
| Day 14 | 0.48 | 0.01 | | 0.58 | 0.001 |
| Day 21 | 0.42 | 0.03 | | 0.69 | <0.0001 |
| Day 28 | 0.39 | 0.04 | | 0.55 | 0.002 |
|  | Intact FGF23 (pg/mL) | | | C-terminal FGF23 (RU/mL) | |
|  | Pearson r | p value | | Pearson r | p value |
| Day 0 | 0.29 | 0.12 | | 0.23 | 0.22 |
| Day 7 | 0.26 | 0.18 | | 0.14 | 0.47 |
| Day 14 | 0.24 | 0.22 | | 0.17 | 0.37 |
| Day 21 | 0.20 | 0.29 | | 0.21 | 0.37 |
| Day 28 | 0.17 | 0.29 | | 0.14 | 0.47 |
