# Supplementary material for: Changes of biomarkers for erythropoiesis, iron metabolism, and FGF23 by supplementation with roxadustat in patients on hemodialysis
Source: Sci Rep. 2023 Feb 23;13:3181. doi: 10.1038/s41598-023-30331-6 (PMC9950357; doi:10.1038/s41598-023-30331-6)
Supplement: Supplementary file 7 — Supplementary Information 7. [file 41598_2023_30331_MOESM7_ESM.docx]

Supplementary table 2. Patient characteristics and laboratory data at baseline (day 0)

|  | Low-ferritin ferritin group | Adequate group | P value |
| --- | --- | --- | --- |
| Number of patients | 14 | 14 | - |
| Age (year) | 74 ± 14 | 75 ± 11 | 0.77 |
| Gender (% of men) | 50 | 36 | 0.44 |
| Body mass index (kg/m^2^) | 22.2 ± 5.2 | 20.1 ± 2.2 | 0.17 |
| Diabetes mellitus (%) | 0 | 29 | 0.05 |
| Kt/V | 1.7 ± 0.2 | 1.7 ± 0.3 | 0.55 |
| Dialysis vintage (months) | 53 (29, 114) | 79 (65, 142) | 0.15 |
| Iron therapy (%) | 64 | 64 | - |
| Intravenous iron (%) | 7 | 36 | 0.07 |
| Oral iron (%) | 57 | 29 | 0.13 |
| Hemoglobin (g/dL) | 11.2 ± 0.1 | 11.0 ± 0.1 | 0.29 |
| Albumin adjusted calcium (mg/dL) | 8.9 ± 0.4 | 9.0 ± 0.5 | 0.42 |
| Phosphate (mg/dL) | 4.5 ± 0.3 | 5.4 ± 0.3 | 0.03 |
| High sensitive C-reactive protein (mg/dL) | 0.08 (0.05, 0.21) | 0.05 (0.05, 0.42) | 0.62 |
| Iron (μg/dL) | 57.0 ± 6.1 | 66.3 ± 6.1 | 0.30 |
| Total iron binding capacity (μg/dL) | 247.3 ± 25.7 | 239.5 ± 22.4 | 0.40 |
| Transferrin saturation (%) | 23.3 ±2.7 | 27.9 ± 2.7 | 0.24 |
| Ferritin (ng/mL) | 58.5 (44.2, 80.1) | 147.0 (132.1, 172.5) | <0.0001 |
| Hepcidin-25 (ng/mL) | 30.1 ± 23.0 | 51.0 ±25.6 | 0.02 |
| Erythropoietin (mIU/mL) | 8.6 (7.3, 13.4) | 10.9 (7.8, 16.4) | 0.49 |
| Erythroferrone (ng/mL) | 0.27 (0.1., 1.27) | 0.37 (0.13, 0.56) | 0.80 |
| GDF-15 (pg/mL) | 4803.1 (3553.3, 5780.9) | 5693.8 (4871.4, 8047.2) | 0.08 |
| Intact PTH (pg/mL) | 187 (155.5, 282.7) | 181 (94.5, 262.5) | 0.56 |
| Intact FGF23 (pg/mL) | 815.5 (306.3, 1186.6) | 1801.2 (945.2, 2260.5) | 0.01 |
| C-terminal FGF23 (RU/mL) | 471.3 162.5, 1101.4) | 873.2 (472.3, 1473.7) | 0.07 |

GDF-15: growth differentiation factor 15, PTH: parathyroid hormone, FGF23: fibroblast growth factor 23
