# Supplementary material for: Changes of biomarkers for erythropoiesis, iron metabolism, and FGF23 by supplementation with roxadustat in patients on hemodialysis
Source: Sci Rep. 2023 Feb 23;13:3181. doi: 10.1038/s41598-023-30331-6 (PMC9950357; doi:10.1038/s41598-023-30331-6)
Supplement: Supplementary file 8 — Supplementary Information 8. [file 41598_2023_30331_MOESM8_ESM.docx]

**Supplementary Figure legends**

Supplementary Figure 1 (Figure S1). Associations between doses of darbepoetin-α and levels of erythropoietin (EPO) (a) or erythroferrone (ERFE) (b), or levels of ERFE and EPO (c) or changes in ERFE and EPO (d) by treatment with darbepoetin-α three days after darbepoetin-α injection. (e) and (f) show associations between levels of ERFE and EPO or changes in ERFE and EPO three days after roxadustat supplementation, respectively. (g) and (h) show associations between levels of ERFE and EPO or changes in ERFE and EPO five days after roxadustat supplementation, respectively. Comparison between EPO concentration three days after darbepoetin-α injection and roxadustat supplementation is shown in Figure S1i.

Supplementary Figure 2. Changes in mean corpuscular volume (MCV) (a), mean corpuscular hemoglobin (MCH) (b), and red blood cell distribution width (RDW) (c). Data are shown as means ± standard deviation.

Supplementary Figure 3. Changes in phosphate, ferritin, and intact and C-terminal fibroblast growth factor 23 (FGF23) grouped by ferritin level with darbepoetin-α injection. Changes in ferritin (a, g), phosphate (b, h), and intact (c, i) and C-terminal FGF-23 (e, k) and % changes of intact (d, j) and C-terminal FGF-23 (f, l) grouped by ferritin <100 (a-f) or ferritin ≥100 ng/mL (g-l) during darbepoetin-α injection. Data are compared between the value at day -7 and the target value. Data are shown as means ± standard deviation. *: p<0.05, †: p<0.01, ‡: p<0.001, §: p<0.0001.

Supplementary Figure 4. Changes in erythropoietin (EPO) (a), erythroferrone (ERFE) (b), growth differentiation factor 15 (GDF15) (c), hepcidin-25 (d), and percentage changes in phosphate grouped by ferritin <100 (closed circles) or ferritin ≥100 ng/mL (open circles) during roxadustat treatment. Data for EPO, EFRE, and GDF15 are shown as medians (interquartile range), and for hepcidin-25 and percentage changes in phosphate are shown as means ± standard deviation. Data are compared between the value at day 0 and the target value. *: p<0.05, †: p<0.01, ‡: p<0.001, §: p<0.0001. **#** indicates a significant difference between the groups.

Supplementary Figure 5. Participant flow diagram and study selection process.
